# Physical Activity and Sedentary Behaviors of Urban Chinese Children: Grade Level Prevalence and Academic Burden Associations

**DOI:** 10.1155/2017/7540147

**Published:** 2017-12-24

**Authors:** Xihe Zhu, Justin A. Haegele, Yan Tang, Xueping Wu

**Affiliations:** ^1^Department of Human Movement Sciences, Old Dominion University, Norfolk, VA 23529, USA; ^2^School of Physical Education and Sport Training, Shanghai University of Sport, Shanghai 200438, China

## Abstract

The objectives of this study were (a) to report grade level prevalence in physical activity and sedentary behaviors and (b) to examine academic burden associations with these behaviors. School-aged children (*n* = 48,118) reported their physical activity, perception of physical activity sufficiency, factors for activity insufficiency, homework hours, and screen time in a typical week. Data were analyzed using general linear models and logistic regression models of Complex Samples. Prevalence results showed that children had lower physical activity and lower screen viewing time, but higher homework time during transition grades (6th, 9th, and 12th) and high school years. Academic burden was cited as the primary reason for not having sufficient physical activity (76.6%). Compared to those citing academic burden, students who did not report academic burden were significantly more likely to meet physical activity guidelines (Odds Ratio (OR) = 5.38, 95% CI = 4.74–6.11), but less likely to meet screen time guidelines (OR = 0.78, 95% CI = 0.72–0.84), controlling for body mass index, gender, and grade level. Additionally, children who reported academic burdens had significantly longer average daily homework time than those who did not (*p* < 0.01). Policy makers should promote physical activity and help children find a balance between homework and physical activity time particularly among the educational transition grades.

## 1. Introduction

Regular engagement in physical activity has been identified as a modifiable lifestyle behavior that can enhance health and decrease the odds of developing chronic diseases (e.g., obesity, cardiovascular disease, and diabetes) throughout the lifespan [1–3]. Physical inactivity is currently identified as the fourth leading risk factor for global mortality [4]. Accordingly, the World Health Organization recommends children to engage in at least 60 minutes of moderate to vigorous physical activity per day [4]. Independent of regular physical activity engagement, sedentary behaviors (e.g., watching television, using computers) can have potentially deleterious health consequences and have been associated with elevated cardiometabolic risk [5–7]. Because of the health issues associated with excessive sedentary behaviors, organizations internationally (e.g., Canadian Society for Exercise Physiology, American Academy of Pediatrics) recommend limiting children's screen time to two hours per day or less [8, 9]. The replacement of sedentary behaviors with moderate intensity physical activity may enhance overall health, as well as assist in preventing chronic disease in youth [10].

It is clear that physical activity and sedentary behaviors are important elements to promote and maintain health, fitness, and well-being [11, 12]. Unfortunately, however, prevalence of children meeting physical activity and screen time guidelines remains low across the globe [13, 14]. In China, while results from the 1997 China Health and Nutrition Survey indicated that youth engaged in significant amounts of physical activity as well as low screen time [15], a more recent cross-sectional study of 1793 youth aged 12 to 15 years in Beijing demonstrated that approximately half (53.6%) of the participants engaged in at least 60 minutes of physical activity daily [16]. Of these participants, many reported exceeding sedentary behavior guidelines and engaging in two or more hours of reading/writing/drawing time (49.7%), computer use time (22.7%), and screen time (42.9%) [16]. For school-aged Chinese children, particularly those living in the urban areas, recent accelerometer-measured physical activity data show that low physical activity and high sedentary behavior engagement are severe issues [17].

Adherence to physical activity and screen time recommendations depend on a number of sociodemographic factors [6, 14, 18]. Namely, younger children, males, those living in more affluent households, and those living in areas with convenient access to outdoor play areas are more likely to meet physical activity guidelines [14, 17]. Older children and those from lower socioeconomic status households are significantly more likely to exceed screen time minimum thresholds [5, 19]. Furthermore, academic burden or stress has been identified as a factor that can impact physical activity, lifestyle, and sedentary behavior in countries with rigorous academic standards and testing [20–23]. Particularly in China, academic burden is greater for higher grade levels, and it is particularly high during educational transition years [20]. For example, a cross-sectional study of 155 middle school students in Beijing (China) demonstrated that having a heavy academic burden such as homework was reported by nearly half (48.4%) of students as the most salient circumstance hindering their engagement in physical activity [21].

In China, youth are under considerable pressure to perform well in school, heavy workloads are typical, and school-aged children tend to spend most free time after school studying [20–23]. Thus, it is reasonable to suggest that academic burden may impact school-aged children's physical activity and sedentary behaviors. Currently, research examining physical activity participation and sedentary behavior habits and related sociodemographic or academic factors among Chinese youth is limited in that previous research is often restricted to convenient samples and/or neglects sedentary behaviors as well as the impact of academic burden [24]. Therefore, the purposes of this investigation were (a) to report grade level prevalence in physical activity and sedentary behaviors and (b) to examine academic burden associations with these behaviors using a large representative sample of school-aged Chinese children in Shanghai. As the only city in mainland China to participate in the Program for International Student Assessment (PISA) tests, which measure the cognitive skills of 15-year-old youth, Shanghai ranked number one in three of the four areas in 2012: mathematics, reading, and science [21]. Compared to other nations, school-aged children living in Shanghai have one of the highest academic burdens and heaviest homework loads internationally [21]. Thus, using a representative sample from Shanghai would provide empirical evidence on this important issue.

## 2. Methods and Materials

### 2.1. Sampling Design and Data Source

In this study, we analyzed data from the 2014 Physical Fitness and Health Index of Child and Adolescents (PFHICA) study. PFHICA used a single-stage, systematic sampling to generate a representative sample of school-aged children in Shanghai, which has the highest urban population in China. School-aged children within schools were randomly selected based on the district and grade level population. A systematic sampling approach was used to ensure each grade level and district population was sampled at about 5%. Student surveys were deployed and conducted in the sampled schools by trained data collectors. For lower grade levels, the questions were read to the participants, and the data collectors assisted filling out the survey, when necessary. The study protocols were approved by Shanghai University of Sport human subjects committee of ethics review board. Written parental consent and child assent forms were obtained for children aged 6–17 years, while written child consent was obtained for students aged 18 years. The data collection processes took place in the fall of 2014, during October and November.

### 2.2. Participants

A total of 50,020 children were invited to participate in the study in 2014. The final sample is composed of 48,118 children (96.2%) from 12 grade levels, 17 municipal counties/districts, and 534 schools that completed the data collection process. The sample is representative of the student population within the grade levels and schools. The children were on average 11.67 (SE = 3.24) years old, ranging from 6 to 18 years. Males and females were equally distributed, with females accounting for 49.5% and male 50.5% of overall weighted population. The participants were predominantly Han ethnic 97.8%. The estimated percentage based on the sample and subgroup count in the sample by gender and grade level are presented in Table 1.

### 2.3. Variables and Measures

#### 2.3.1. Physical Activity

Two questions adopted from Active Healthy Global Alliance study were used to assess children's physical activity [25]. For the first question, they were asked, “During the previous five week days, on how many days were you physically active for a total of at least 60 minutes per day?” The children selected a response ranging from zero days to five days. For the second question, they were asked, “During the previous two weekend days, on how many days were you physically active for a total of at least 60 minutes per day?” The children selected a response ranging from zero days to two days. We aggregated the responses from these two questions to compute the total number of days that children were physically active for at least 60 minutes. We also calculated the overall percentage of students who met the recommended guidelines of being physically active for at least 60 minutes daily. Additionally, children were surveyed about whether they felt they had sufficient physical activity (yes/no) and factors that contributed to physical activity insufficiency. The listed factors included (a) no time due to academic burden, (b) no interest in physical activity, (c) lack of sport skills, (d) lack of parent support, (e) health problems, (f) lack of space/equipment, and (g) other.

#### 2.3.2. Sedentary Behaviors

We categorized children's sedentary behavior time into two broad categories: screen time and homework time. The survey questions measuring these behaviors were adapted from Active Healthy Global Alliance study [25]. For screen time, children were asked about their time spent engaged with TV/movies, video games, computers, and electronic devices. Three pairs (one focusing on week day and one focusing on weekend day) of questions included the following: (1) “Over the previous week (weekend) days, on average how many hours per day did you sit and watch TV, movies, or videos?” (2) “Over the previous week (weekend) days, on average how many hours per day did you play video, computer, or other electronic games outside of school?” and (3) “Over the previous week (weekend) days, on average how many hours per day did you sit and use computers and other electronic devices to conduct the following activities including instant messaging, web browsing, checking emails, etc.?” Possible responses to these three pairs of questions included the following categories: none, about half hour, 1 hour, 2 hours, 3 hours or more. Children's screen time average (hours/days) was derived by the weighted aggregate of the responses from these three pairs of questions.

Children were asked to respond to two questions about their homework time. For the first question, they were asked “During the week days, how many hours do you usually spend doing your homework afterschool?” The second question stated “During the weekend days, how many hours do you usually spend doing your homework afterschool?” The response options for both questions were none, about half hour per day, 1 hour per day, 2 hours per day, 3 hours or more per day. Children's homework time mean (hours/days) was derived from the weighted aggregate of the responses from these two questions.

#### 2.3.3. Demographic Variables

Recorded demographic variables included age, ethnicity, gender, and grade level. Grade levels span from Grade 1 to Grade 12 in Shanghai, with the first nine years being compulsory by law, including six years at elementary and three years at middle school levels. The last three years are either predominantly high school or vocational school. Grades six, nine, and twelve are educational transition years when children will be systematically tested and evaluated for advancing to middle school, high school, and college, respectively. Students are often faced with higher academic burden during these transition years.

### 2.4. Statistical Analysis

We conducted data analyses using Complex Samples in SPSS (version 22, IBM, Armonk, NY, USA) taking into account the stratified sampling plan for statistical estimates. Prevalence for demographic variables as well as children meeting physical activity and screen time recommendations was computed from the Complex Samples to account for the sampling plan. Comparison of proportions was estimated using Pearson likelihood ratio test. To estimate and examine grade level prevalence in children's physical activity, screen time, and homework time, we conducted general linear models (GLM) with children gender, grade level, and their interaction term as independent variables, controlling for their body weight index. To examine the association between academic burden, physical activity, and sedentary behaviors, we conducted logistic regressions in Complex Samples to test the Odds Ratio (OR) differences in meeting physical activity guidelines of one hour per day and screen time guidelines of less than two hours per day, between those reporting academic burden and those not, controlling for children grade level, gender, and body mass index. Confidence intervals of 95% for OR were also reported. Additionally, to test the association of academic burden with homework time, we ran a separate GLM to compare homework time between those reporting academic burden and those not, controlling for gender, grade level, and body mass index. Tests were considered statistically significant using an overall *p* < 0.05.

## 3. Results

### 3.1. Grade Level Prevalence and Variation

On average, children reported 3.39 (SE = .05) days/week where they engaged in physical activity time for one hour or more, 1.60 (SE = .03) hours/days screen time, and 1.76 (SE = .03) hours/day homework time. Overall, 20.0% (SE = 3.6) of children met the recommended physical activity of 1 hour or more daily, and 73.5% (SE = 1.5) of children met screen time recommendations of less than two hours per day. However, 63.3% (SE = 1.2) of children felt that they engaged in sufficient amounts of physical activity, and 36.7% (SE = 1.2) felt they engaged in an insufficient amount. Those who felt that they had insufficient physical activity commonly cited no spare time due to academic burden 76.6% (SE = 1.5) and a lack of space/equipment 49.6% (SE = .9) as contributing factors. Other factors were much less frequently cited as contributing factors for physical activity insufficiency: no interest in physical activity 21.0% (SE = .5), lack of sport skills 18.8% (SE = .4), lack of parent support 4.1% (SE = .1), health problems 5.4% (SE = .1), and other 6.2% (SE = .1). Because the participants could check more than one reason for not having sufficient physical activity, these percentiles do not add up to 100%.

**Figure 1:**
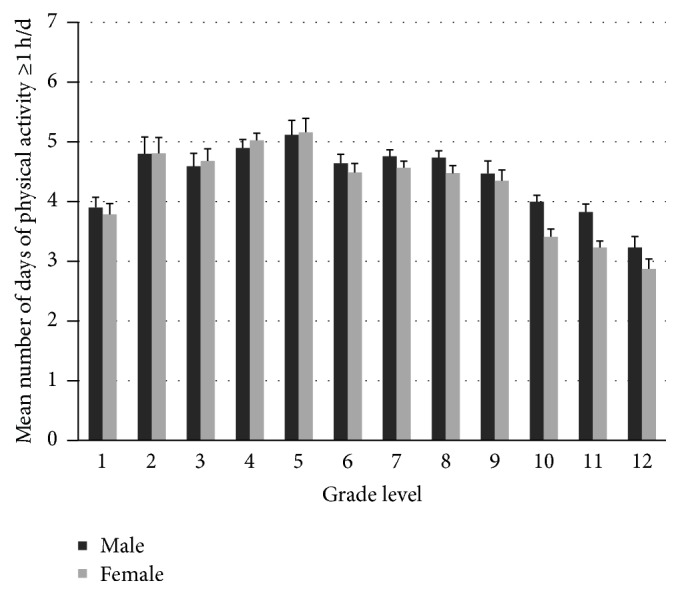
Mean number of days per week (d/wk) in which children are physically active for 60 minutes or more.

GLM results showed that gender, grade, and their interaction gender *∗* grade were significantly associated with the mean physical activity days per week (Wald *F*_24,510_ = 22.36, *r*^2^ = 0.082, effect size *f*^2^ = 0.09, *p* < 0.001). In general, female participants had a lower mean number of physical activity days than males (*β* = −0.15, *F*_1,533_ = 18.27, *p* = 0.001). As shown in Figure 1, the mean number of days that children were physically active for one hour or more did not differ significantly between males and females in grades 1 through 9. Females reported significantly lower number of days per week than males for grades 10 to 12 (*β* ≤ −0.37, *t* = −3.29, *p* = 0.001). There were significant differences in the number of days among different grades (Wald *F*_11,523_ = 18.15, *p* < 0.001); specifically, grades 2, 4, and 5 had significantly higher days than grades 1, 3, 6,7, 8, and 9, which were higher than grades 10, 11, and 12.

**Figure 2:**
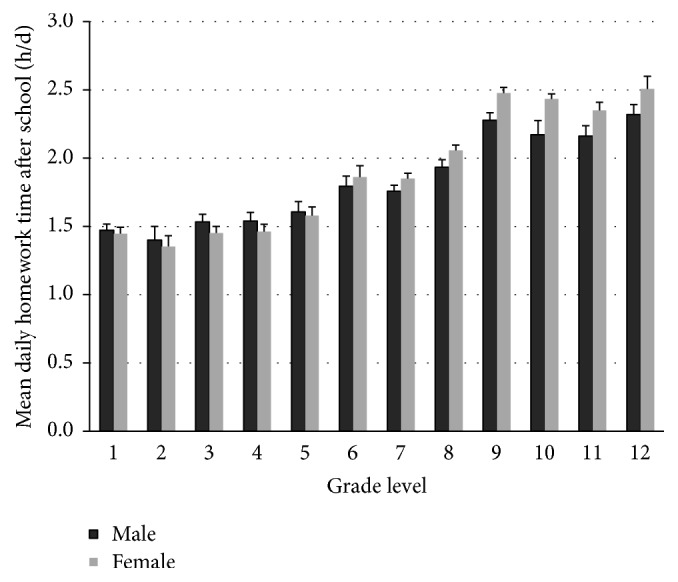
Children's mean daily homework time (h/d) after school.

Gender, grade, and their interaction were significantly associated with mean daily homework time (Wald *F*_24,510_ = 51.22, *r*^2^ = 0.18, effect size *f*^2^ = 0.22, *p* < 0.001), explaining about 17.6% of variance in student homework time. As shown in Figure 2, children in grade levels 9 through 12 reported significantly higher amounts of homework time than those in grades 1 to 5 (*β* ≤ −0.22, *t* = −1.98, *p* = 0.04). While males reported slightly higher homework time during grades 1 to 5, it is unique that females reported significantly higher daily average homework hours than males throughout grades 9 to 12 (*p* < 0.001).

**Figure 3:**
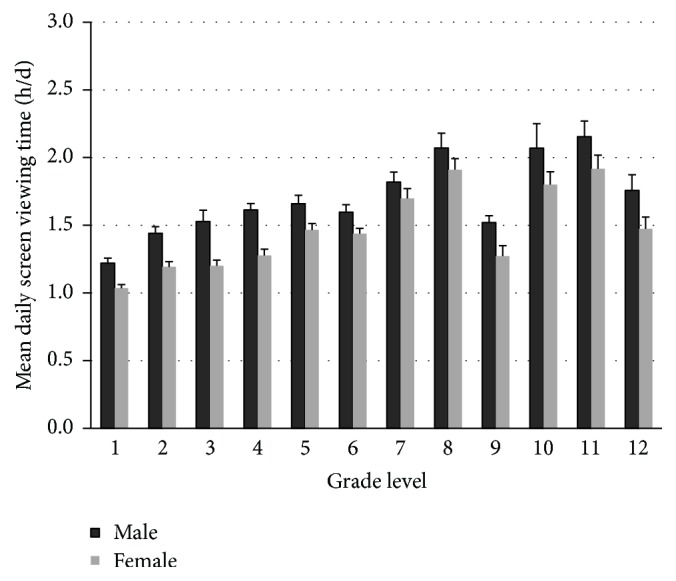
Children's mean daily screen viewing time (h/d).

Children's daily mean screen time after school is presented in Figure 3. The results from GLM suggest that there are significant gender (Wald *F*_1,533_ = 68.12, *p* < 0.001) and grade (Wald *F*_11,523_ = 28.97, *p* = 0.001) associations, but there is no significant interaction (Wald *F*_11,523_ = 4.10, *p* = 0.625). On average, males reported higher daily screen time than females across all 12 grade levels (*β* = .19, *F*_1,533_ = 68.17, *p* < 0.001). As seen in Figure 3, although children in higher grade levels reported higher daily screen time than those in lower grades in general, there were exceptions in that children in grade 9 had significantly lower screen time than those in grades 7 and 8 (*β* ≤ −.12, *p* ≤ 0.03), and children in grade 12 reported significantly lower screen time than their counterparts in grades 10 and 11 (*β* =* 0.23*, *p* = 0.028). Coincidentally, there were spikes in children's homework time at grades 9 and 12 (Figure 2).

### 3.2. Association with Academic Burden

Children's reported academic burden was associated with their odds of meeting physical activity guideline of one hour per day and the odds of meeting screen viewing time guideline of less than two hours per day. As seen in Table 2, compared with children who reported academic burden, those who did not were about five times more likely to meet physical activity guidelines (OR = 5.38, 95% CI: 4.74–6.11, *p* < 0.01) but were less likely to meet the screen viewing time guidelines (OR = 0.78, 95% CI: 0.72–0.84, *p* < 0.01), controlling for participant body mass index, gender, and grade level. Additionally, separate GLM results showed that children who reported academic burden had significantly higher daily average homework time (*F* = 1190.49, *f*^2^ = 0.22, *β* = 0.39, *p* < 0.01), with those reporting academic burden averaging 2.08 ± .05 h/d and those not 1.69 ± .05 h/d, holding body mass index, gender, and grade level constant.

## 4. Discussion

The purposes of this study were (a) to report grade level prevalence in physical activity and sedentary behaviors and (b) to examine academic burden associations with these behaviors among a representative sample of school-aged Chinese children in Shanghai. For physical activity participation, while the findings are in general consistent with previous reports in that males are more physically active than females [14, 17], this study adds new findings to the literature suggesting that the significant differences between them seemed to appear during 10–12th grade levels. Children's physical activity days of more than 1 hour/day in relation to grade level form a second-order polynomial trend line peaked at late elementary years and valleyed at 12th grade (Figure 1). This grade level prevalence and variation add to our understanding of a recent national prevalence study [24]. During major educational transition grades (e.g., 6th, 9th, and 12th grades) and high school years when academic burden is high [20, 22], academic burden is cited by a significantly higher percentage of the children as the reason for not having sufficient physical activity time. More importantly, logistic regression results showed that those who did not report academic burden were about five times more likely to meet physical activity guidelines than those who reported that, holding body mass index, gender, and grade levels constant. This finding suggests a significant negative impact of academic burden on children's physical activity participation [21].

Those who reported academic burden tended to have longer homework time, controlling for body mass index, gender, and grade level. While the overall mean homework hours in this study are comparable with other reports of around 11 hours per week [16, 21, 22], within the same grade cohort (e.g., elementary or middle) sixth and ninth graders (transition grades) reported significantly higher homework hours than other grades (Figure 2). The interaction between gender and grade is a unique finding, revealing that males and females report similar homework hours until the late middle school years, after which females report significantly higher homework hours than males (Figure 2). This result may help to explain the on-going trend of gender inequality reversal in higher education [26].

Due to economic and technological advancements, school-aged Chinese children in this study (surveyed in 2014) reported higher amounts of screen time than reported in an earlier 1997 survey [15], and this finding is generally consistent with a recent national estimate [24], although their prevalence of high screen time (>2 h/d) is still lower than some Western peers [5]. As seen in Figure 3, the difference between gender and grade is apparent in that children in higher grade levels tend to report higher screen time and males tend to report higher screen time than girls across grade levels. As discussed earlier, the academic burden during the educational transition years has a significant association with screen time as well, which is very telling among children in 9th and 12th grades as their screen time is significantly lower than the other grades in the same cohorts (Figure 3). Interestingly, children who did not report academic burden were less likely to meet screen viewing time guidelines than those who did. In other words, reporting of academic burden tends to be positively associated with lower screen viewing time.

Limitations of the study include using self-reported data on physical activity and sedentary behaviors including homework and screen viewing time. Because the data were collected during the fall season, the results of this study may not fully capture physical activity and sedentary behaviors which occur during other seasons. Additionally, the multiple choice format in the survey may lead to information bias of the study and limits estimation of the sedentary behavior time and interpretation of the report. For example, the choices for sedentary behavior time limited to an upper bound of three hours, which could limit the approximation of either screen viewing time or homework time for certain groups of children. Finally, lifestyle-related variables were not taken into account in this study, and future studies should consider adding them as covariates [27].

## 5. Conclusion

In summary, the findings in this study added to our understanding of grade level prevalence and variation in children' physical activity and sedentary behaviors [5, 16, 17, 24] and of academic burden that was cited as an important factor but was not closely examined by other studies [15, 21]. The findings further revealed that while those reporting academic burden were less likely to meet physical activity and screen viewing time guidelines than their counterparts, they tended to have longer homework time after school. These findings suggest that policy makers in China should focus on promoting physical activity and help parents and children find a balance between homework and physical activity time. Physical activity rarely negatively impacts or may be positively related to academic achievement [28], and physical activity may be helpful to alleviate stress due to academic burden [29], particularly during the transition grades. Physical education curriculum can help improve children's knowledge and values about physical activity in schools [30]. Results from this study also suggest that females need more support for physical activity participation and perhaps relieving academic burden than males during high school years.

## Figures and Tables

**Table 1:** Demographic characteristics and sample size for analyses (Shanghai, China 2014).

Grade	Gender	Weighted%^‡^	95% CI	Subgroup count
1	Male	52.0%	50.8%–53.2%	1719
	Female	48.0%	46.8%–49.2%	1587
2	Male	51.2%	49.8%–52.6%	2381
	Female	48.8%	47.4%–50.2%	2270
3	Male	51.1%	49.7%–52.6%	2409
	Female	48.9%	47.4%–50.3%	2301
4	Male	51.6%	50.1%–53.1%	2745
	Female	48.4%	46.9%–49.9%	2578
5	Male	51.9%	51.2%–52.6%	2808
	Female	48.1%	47.4%–48.8%	2603
6	Male	51.4%	49.7%–53.2%	2017
	Female	48.6%	46.8%–50.3%	1905
7	Male	48.5%	46.9%–50.1%	2408
	Female	51.5%	49.9%–53.1%	2559
8	Male	49.7%	48.9%–50.5%	2466
	Female	50.3%	49.5%–51.1%	2498
9	Male	49.8%	46.6%–53.0%	1229
	Female	50.2%	47.0%–53.4%	1241
10	Male	49.4%	46.6%–52.2%	2011
	Female	50.6%	47.8%–53.4%	2059
11	Male	48.0%	45.8%–50.2%	1586
	Female	52.0%	49.8%–54.2%	1718
12	Male	48.5%	45.0%–52.0%	1124
	Female	51.5%	48.0%–55.0%	1194

*Note.* Sample size *N* = *48,118*. ^‡^Percentages are weighted according to student populations by grade level and schools in Shanghai.

**Table 2:** Odds Ratio of meeting physical activity and screen viewing time guidelines (Shanghai, China 2014).

	*B*	SE_*B*_	*t*	*p*	Odds Ratio (95% CI *e*^*B*^)
Model 1: physical activity time guideline^a^ (*F*= 28.79, Pseudo *R*^2^ = 0.11, *p*< 0.01)					

Reported academic burden	Reference				
Not reported	1.68	0.06	28.08	0.00	5.38 (4.74–6.11)

Model 2: screen viewing time guideline^b^ (*F*= 55.69, Pseudo *R*^2^ = 0.04, *p*< 0.01)					

Reported academic burden	Reference				
Not reported	−0.25	0.03	−7.46	0.00	0.78 (0.72–0.84)

*Note.* Covariates include participant body mass index, gender, and grade level. *e*^*B*^ = exponentiated *B*. ^a^Reference category: not meeting recommended physical activity for at least one hour per day. ^b^Reference category: not meeting recommended screen viewing time of less than two hours per day.
